# Synthesis and Characterization of Calcium Sulfoaluminate Hydrates—Ettringite (AFt) and Monosulfate (AFm)

**DOI:** 10.3390/ma17215216

**Published:** 2024-10-26

**Authors:** Wojciech Szudek, Jakub Szydłowski, Ilona Buchała, Ewa Kapeluszna

**Affiliations:** Department of Building Materials Technology, Faculty of Materials Science and Ceramics, AGH University of Krakow, Al. Mickiewicza 30, 30-059 Kraków, Poland; szydlows@agh.edu.pl (J.S.);

**Keywords:** ettringite, monosulfate, calcium sulfoaluminate hydrates, synthesis, AFt, AFm

## Abstract

The goal of the presented work was to find the most favorable conditions for the synthesis and stabilization of chemically pure ettringite and monosulfate. The reaction was carried out by mixing pure tricalcium aluminate (C_3_A) and gypsum (C$\bar{S}$H_2_) in an excess amount of water. The impact of hydration time (2–7 days), C_3_A:C$\bar{S}$ molar ratio (1:1–1:3) and water vapor pressure of the selected drying agents (anhydrite-III and supersaturated CaCl_2_ solution) on the phase composition of the products was evaluated. After 7 days of hydration, either ettringite or monosulfate was obtained as the main product, depending on the C_3_A:C$\bar{S}$ molar ratio. The synthesis carried out at a C_3_A:C$\bar{S}$ molar ratio of 1:3 produced pure ettringite. In the case of the sample characterized by the ratio of 1:1 (typical of monosulfate), a considerable portion of ettringite (27.9%) was present in the final products along the AFm phase. Therefore, a different synthesis method has to be selected in order to obtain pure monosulfate. The results showed that thermal analysis, X-ray diffractometry and FTIR spectroscopy can be used to distinguish the characteristic features of ettringite and monosulfate.

## 1. Introduction

### 1.1. Calcium Sulfoaluminate Hydrates in Ordinary Portland Cement (OPC) Systems

In Portland cement systems, calcium sulfoaluminate hydrates start to form in the very first minutes of hydration as a result of the reaction of tricalcium aluminate (C_3_A) and calcium aluminoferrite (brownmillerite—C_4_AF) with water in the presence of sulfate ions (released by the so-called “set regulator”, typically gypsum—C$\bar{S}$H_2_ or anhydrite—C$\bar{S}$) [1]. In the absence of calcium sulfate, C_3_A reacts rapidly with water, leading to an almost instantaneous cement setting. Such an outcome is undesirable, as good workability is required in order to place and consolidate the concrete [2,3].

In the presence of sulfate ions, the C_3_A hydration rate decreases considerably after a short period of rapid reaction, during which ettringite (AFt—C_6_A$\bar{S}$_3_H_32_) is produced as the main phase [2]:

$$C_{3}A+3\;C\bar{S}+32\;H\;\to \;C_{6}A{\bar{S}}_{3}H_{32}$$

For decades, the literature explained the cause of this deceleration effect as the formation of a tight ettringite layer on the surface of tricalcium aluminate grains that creates a diffusion barrier and seals them off [2,3,4]. Recently, however, several papers found the adsorption of sulfate ions on the reactive dissolution sites of C_3_A to be a more probable mechanism [2,4,5]. Nevertheless, once C$\bar{S}$ has all been consumed, the hydration rate increases, with calcium aluminate monosulfate (AFm—C_4_A$\bar{S}$H_12_) being formed as the main product at the expense of ettringite [2,4]:

$$2\;C_{3}A+3\;C_{6}A{\bar{S}}_{3}H_{32}+4\;H\;\to \;3\;C_{4}A\bar{S}H_{12}$$

The crystallization of the so-called “primary ettringite” takes place in the early stage of cement hydration. However, in the case of hardened concretes, under certain conditions delayed ettringite formation (DEF) might occur [6]. The phenomenon is one of the most dangerous and harmful corrosion mechanisms, as the expansive nature of the phase leads to severe cracking, especially in the case of constructions exposed to wet environments [7,8].

### 1.2. Delayed Ettringite Formation

In the past few decades, numerous studies have been dedicated to explaining the mechanism of delayed ettringite formation in hardened concretes. A thorough review was published by Taylor et al. [6], according to which there are two necessary but insufficient conditions for DEF expansion to occur. First, the internal temperature of the composite in the early stage of hydration must exceed 70 °C for a sufficiently long time. Second, after the temperature returns to an ambient level, the material must be kept wet or moist intermittently or permanently. Furthermore, the expansion occurs more easily in mortars and concretes prone to alkali–silica reaction (ASR), as ettringite tends to crystallize in the cracks formed at the aggregate–paste interface as a result of ASR [6,9]. However, the authors considered DEF in terms of an internal process in which none of the sulfate ions come from the outside environment. Collepardi [8], on the other hand, reviewed the literature considering delayed ettringite formation due to both internal and external sulfate attacks and came to the conclusion that the external attack, occurring due to the absorption and diffusion of sulfates from wet environments, can only be prevented by the use of impermeable concrete. Meanwhile, the internal attack is caused by the late release of SO_4_^2−^ ions from cement, C-S-H phase or, very rarely, gypsum-contaminated aggregate. Regardless of the mechanism, the risk of DEF-related composite damage can be prevented by precluding either one of the following factors: microcracking, delayed sulfate release or water exposure. The phenomenon of the adsorption of sulfate ions on C-S-H gel was also studied by Fu et al. [10]. The authors determined that the effect intensifies when the internal temperature of the composite is growing; then, when the system has cooled down, the ions are slowly desorbed, affecting the composition of the pore solution and leading to ettringite crystallization.

In one of the first papers dedicated to the matter, Mehta [11] postulated that in Portland cement composites, ettringite can be present both as large, needle-like crystals and in colloidal form, depending on the amount of lime in the system, and that only the latter is capable of developing large expansions. However, an outside source of water is required for the effect to occur. Scherer [12,13] analyzed the ettringite recrystallization phenomenon, determining that if a high temperature is maintained in the initial curing period, its subsequent decrease leads to supersaturation of the pore solution. The capillary pores of various sizes found in cement composites form a network. Once the voids are filled with water, small ettringite crystals are dissolved. Due to a concentration gradient, the ions released in the process are diffused towards bigger pores, where they contribute to the further growth of larger crystals, consistent with the so-called Ostwald ripening. However, the formation of large ettringite needles does not necessarily have to be deleterious. On the contrary, they often only fill in the voids formed as a result of other harmful processes [6,12,13,14]. Müllauer et al. [15] determined that during sulfate attack, the formation of ettringite crystals (mainly from AFm) in small, 10–50 nm pores generates a stress of approx. 8 MPa, exceeding the tensile strength of the cementitious matrix (3 to 4 MPa) and thus leading to expansion and cracking. Meanwhile, the crystallization of ettringite in larger pores is not deleterious, as the generated pressure is insufficient to damage the matrix.

Several studies, e.g., by Nguyen et al. [16], showed that partial substitution of Portland cement with fine pozzolans limits or even completely eliminates the phenomenon of delayed ettringite formation in OPC composites. Such an effect is a consequence of the decreased porosity and, therefore, lower capillary absorption of mortars and concretes, combined with a decrease in the Ca(OH)_2_ content. Highly reactive pozzolans have the most pronounced effect [17]. On the other hand, coarser mineral additives act in an opposite way, leading to the formation of higher amounts of ettringite. Lothenbach et al. [18] reported that when calcium carbonate is used as a cement additive, the transition of ettringite to monosulfate is prevented, as the latter is rendered unstable. Instead, calcium mono- and hemicarboaluminate are formed, which indirectly stabilize the ettringite. Furthermore, De Weerdt et al. [19] determined that the effect is more pronounced and even higher amounts of AFm/AFt phases are found when fly ash is additionally present in the system.

### 1.3. Calcium Sulfoaluminate (CSA) and Belite-Ye’elimite-Ferrite (BYF) Cement Systems

In recent years, as part of the ongoing search for more sustainable alternatives to Portland cements, calcium sulfoaluminate (CSA) and belite-ye’elimite-ferrite (BYF) cements have gained increased scientific attention [20,21]. The most effective clinkering temperature for CSA and BYF clinkers is approx. 1250 °C, which is 200 °C lower than the temperature used for Portland cement clinker (1450 °C) [20,22]. According to Ben Haha et al. [20], considering various heat losses along the production line, the overall reduction in the heat of clinkering can be estimated at 10–15%. Furthermore, in the case of CSA cements, the amount of limestone used in the raw material mix is 35–40% lower compared to OPC [22]. As a result, the CO_2_ emissions associated with the manufacturing of BYF and CSA clinkers are approx. 20–35% lower [20]. Based on the stoichiometry of the chemical reactions involved, Tao et al. [23] calculated that 0.54 tons of CO_2_ is released during the production of 1 ton of Portland clinker, while in the case of 1 ton of CSA clinker, the CO_2_ emissions are reduced to only 0.27 tons. Therefore, in comparison with OPC, the carbon footprint of cementitious composites manufactured with the use of CSA and BYF cements is considerably lower [21]. CSA cements are also characterized by favorable engineering properties, such as fast setting and hardening, which makes them suitable for urgent repairing, sealing and soil stabilization [23]. Recent studies [24] proved that apart from high compressive strength, CSA concretes feature good durability in corrosive environments, such as marine tidal zones. Moreover, research on CSA/OPC blends shows promising results, as such binders combine the advantages of both cements, providing fast to rapid setting and hardening and high early and late compressive strength [25,26,27,28]. Thus, they can be used as a sustainable solution for novel applications, such as 3D-printing [29].

The behavior of CSA cements is regulated mainly by ettringite, formed upon the hydration of their main mineralogical component, ye’elimite (C_4_A_3_$\bar{S}$), in the presence of calcium sulfate:

$$C_{4}A_{3}\bar{S}+2\;C\bar{S}+38\;H\;\to \;C_{6}A{\bar{S}}_{3}H_{32}\;+\;2\;{\mathrm{AH}}_{3}$$

Since the stoichiometry of ye’elimite is aluminum rich with respect to ettringite, microcrystalline AH_3_ is formed as a by-product. If a calcium sulfate source is absent, ye’elimite hydration yields monosulfate (C_4_A$\bar{S}$H_12_) instead of ettringite [30]:

$$C_{4}A_{3}\bar{S}+18\;H\;\to \;C_{4}A\bar{S}H_{12}\;+\;2\;{\mathrm{AH}}_{3}$$

Ettringite is characterized by an early onset of its mechanical strength and, under certain conditions, its crystallization is associated with considerable expansion, capable of developing shrinkage compensation in the matrix [31]. Therefore, the kinetics of ettringite formation regulate the mechanical properties of composites containing CSA and BYF cements. Pure ye’elimite is often used as a model system for CSA and BYF dissolution [20]. In a similar manner, synthetic ettringite and monosulfate can be used in research as model hydration products. Thus, it is of great scientific importance to find an easy and efficient process to obtain pure AFm and AFt phases.

### 1.4. Synthesis of Calcium Sulfoaluminate Hydrates

In the literature, several ettringite synthesis methods have been described. One, reported by, among others, Kishar et al. [32,33], is based on the reaction of pure tricalcium aluminate with gypsum in an excess amount of water. The raw materials were mixed at a molar ratio of 1:1. The synthesis was carried out for 30 min and 3, 14, 28, 60 and 90 days. Following these hydration periods, the samples were subjected to an XRD analysis, the results of which are presented in Table 1.

The authors determined that after 30 min of hydration, the reaction degree of the system was very low—mostly C$\bar{S}$H_2_ and C_3_A were present in the samples, accompanied by only small amounts of ettringite. After 3 days, the specimens contained significantly more ettringite but also unreacted gypsum and tricalcium aluminate, while after 14 days at 30 °C, only traces of C_3_A were found in the XRD pattern. Later on, the formation of monosulfate was observed. However, after 60 or 90 days of hydration (depending on the reaction temperature), recrystallization of ettringite took place, proving that monosulfate is indeed not a stable phase at either 30 or 50 °C.

Another way to synthesize ettringite is through the hydration of synthetic ye’elimite, analogously to its naturally occurring formation process. The method was used in a study by Gemrich et al. [34]. The XRD analysis presented by the authors proves that after 6 h of C_4_A_3_$\bar{S}$ hydration, the sample consisted mostly of ettringite. After 7 days, reflections characteristic of either C_4_AH_13_ or monosulfate started to show in the diffractograms; however, the authors did not differentiate between the two. The biggest downside of this synthesis method is that even after 14 and 21 days of hydration, small amounts of other phases—ye’elimite, calcite and monosulfate—were found in the samples along with ettringite. Therefore, when pure phase needs to be obtained, a more suitable synthesis method has to be selected.

In the prior literature, an often-used (e.g., by Terai et al. [35]) ettringite synthesis technique is based on the following reaction:

$$4\;\mathrm{CH}+A{\bar{S}}_{3}H_{18}+10\;H\;\to \;C_{6}A{\bar{S}}_{3}H_{32}$$

The method is popular as it requires cheap and widely available reagents. The synthesis can be carried out in one of two ways—by dissolving aluminum sulfate hydrate in water and mixing it with a stoichiometric amount of calcium hydroxide or by grinding the raw materials together and hydrating the obtained homogenic powder. In the cited study, the authors aimed to determine the influence of the Ca/Al molar ratio on the phase composition of the reaction products. According to the results, the ratios of 4.0 and 4.5 are the most favorable, as they lead to the formation of pure ettringite. In all other cases, portlandite, calcite and gypsum were present among the synthesis products. Such an outcome was surprising, as the most ettringite was expected at the stoichiometric ratio of 3.0.

During the synthesis of ettringite, appropriate drying conditions have to be applied in order to maintain the morphology and stability of the phase—the process should only remove free, chemically unbound water. Luo et al. [36] studied the impact of several drying methods on the crystal structure of synthetic ettringite. In their research, the authors used vacuum, oven and D-drying, with and without pretreatment in various organic solvents (acetone, ethanol and isopropanol). The results showed that vacuum drying at room temperature preserves the most ettringite—only reflections characteristic of AFt and gypsum were found in the XRD pattern of this particular sample, while the diffractograms of the oven-dried specimens revealed the additional presence of monosulfate. D-drying led to the complete decomposition of ettringite, while the use of organic solvents changed its unit cell parameters and made it prone to carbonation. Similar conclusions were drawn from the results of the thermal analysis. Moreover, SEM observations showed that in the case of vacuum drying, the crystals were the longest and the most well formed.

Khoshnazar et al. [37] investigated the influence of three hydration stoppage methods—through solvent exchange by methanol, ethanol and isopropanol—on the stability of pure ettringite. XRD analysis showed that when immersed in methanol for 24 h, the ettringite was completely decomposed. Even a short, 15-min treatment led to a significant decrease in the intensity of the peaks observed in the diffractograms. According to the authors, methanol molecules are small (diameter of 3.6 Å) and therefore, apart from surface adsorption, they are able to enter the intercolumnar space of the ettringite structure and partially replace the water molecules in the main vertices, resulting in a considerable distortion of the crystals. The theory was additionally supported by the FTIR spectroscopy results. Meanwhile, the XRD patterns of the specimens treated with ethanol and isopropanol showed no major alterations compared to the reference sample. Similar conclusions were drawn from the results of the thermal analysis.

Renaudin et al. [38] compared the structural characteristics of two ettringite samples—the first one was obtained straight from the synthesis liquid, while the second was dried to 35% relative humidity over a saturated CaCl_2_ solution. Crystallographic studies proved that the structures of both specimens were similar—small differences were observed in the values of *a* and *c* unit cell parameters, but no other modifications were found and the amount of channel water remained intact. The same conclusions were drawn from the results obtained by Raman spectroscopy. Therefore, the authors declared that the selected drying technique preserves the morphology and structure of synthetic ettringite.

A very important issue regarding the synthesis of ettringite and, especially, monosulfate, is their protection from carbonation. At a temperature of 20 °C, when CO_3_^2−^ ions are present, the transition of AFt to AFm is hindered. Instead, carboaluminate and ettringite become the two stable phases [18,39]. Carbonate ions, formed due to the absorption of in-air CO_2_, can easily substitute sulfates in the structure of the AFm phase, leading to the formation of solid solutions [3]. Therefore, in order to obtain chemically pure, “uncontaminated” monosulfate, the samples need to be stored in sealed containers and their contact with in-air CO_2_ must be kept to a minimum.

### 1.5. Research Objectives

Despite the fact that a great deal of research on calcium sulfoaluminate hydrates has been carried out over the last couple of years, numerous questions remain unanswered about the impact of different chemical and physical factors on their structure and stability. The growing awareness of the need for climate protection and the continuous attempts to lower the CO_2_ emissions associated with cement manufacturing have led to a global search for new, more sustainable solutions for cementitious composites. One way to approach the issue is to substitute clinker with alternative mineral additives, especially in the form of industrial by-products [40,41,42,43,44,45,46,47,48,49]. Another is to use cements other than OPC, such as CSA or BYF, the hydration of which is associated with the formation of large amounts of ettringite. Therefore, performing further studies on the durability and stability of calcium sulfoaluminate hydrates, especially ettringite and monosulfate, is extremely relevant from a scientific and practical point of view. However, before such research can be carried out, the most favorable conditions have to be determined that allow for the synthesis of chemically pure AFt/AFm, which was the main goal of the presented work. Additionally, the study aimed to characterize the obtained phases with the use of X-ray and thermal analyses and FTIR spectroscopy.

## 2. Materials and Methods

X-ray diffractograms were collected in a 2θ range of 5–65° using a PANalytical Empyrean diffractometer (Malvern, UK) equipped with a Cu/Ni lamp operating at 35 kV/16 mA. Step size was 0.05 °2θ, scan speed 4 s/step. The phase composition of the samples was analyzed using X’Pert Highscore Plus software (ver 5.1, ICSD database). DTA/TGA measurements were performed in the temperature range of 25–1000 °C with a Netzsch STA 449F3 Jupiter thermoanalyzer (Selb, Germany). A synthetic air atmosphere and a gas flow of 40 mL/min were used. FTIR spectra were collected with a Bruker Vertex 70v vacuum spectrometer (Billerica, MA, USA) using a transmission technique in the mid-infrared. The tablet method was applied (KBr, Merck, Darmstadt, Germany). For each spectrum, 128 scans were recorded in the range of 4000–400 cm^−1^ at a resolution of 2 cm^−1^.

**Figure 1:**
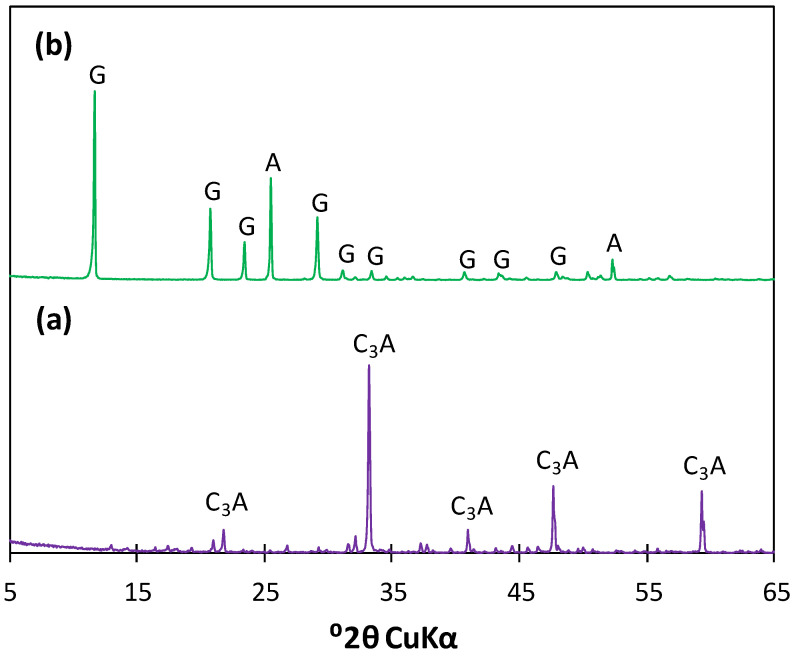
Diffractograms of raw materials used in the synthesis of calcium sulfoaluminate hydrates: (**a**) C_3_A and (**b**) C$\bar{S}$H_2_; **G**—gypsum, **A**—anhydrite.

**Figure 2:**
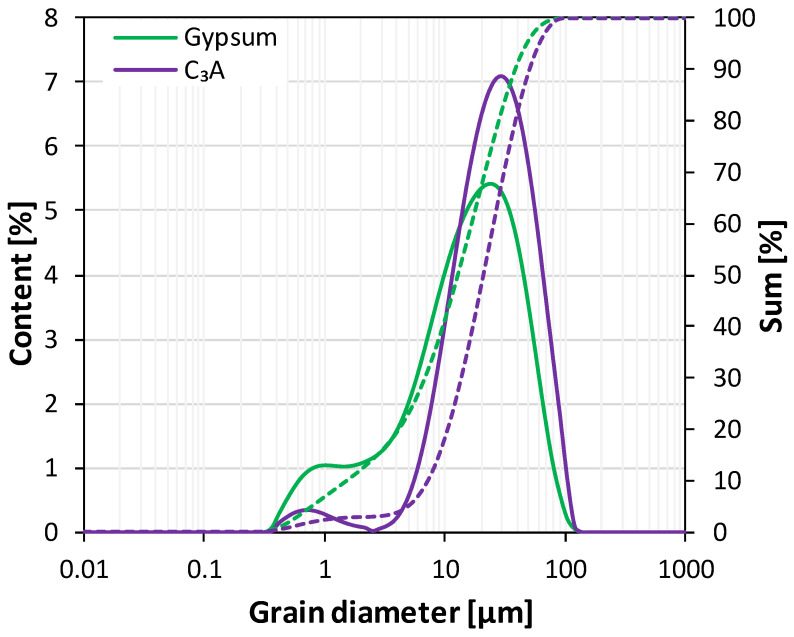
Particle size distribution of the raw materials used in the study; solid lines—content [%], dotted lines—sum [%].

The synthesis was carried out by mixing pure synthetic tricalcium aluminate (C_3_A) and gypsum (C$\bar{S}$H_2_) in an excess amount of water. Tricalcium aluminate (characterized by a cubic crystal system) was synthesized from analytical grade calcium carbonate (CaCO_3_, POCH S.A., Gliwice, Poland) and aluminum oxide (Al_2_O_3_, POCH S.A.) mixed in stoichiometric proportions. The powders were homogenized and pressed into pellets, then sintered twice at 1400 °C. The XRD analysis (Figure 1a) proved that the obtained C_3_A was of high purity—only reflections characteristic of tricalcium aluminate were observed (d_hkl_ = 2.69, 2.21, 1.81) and no traces of hydration products were found that would indicate a partial reaction of the phase prior to the experiment. Analytical grade gypsum (CaSO_4_·2H_2_O, POCH S.A.) was used as the calcium sulfate source. High-intensity peaks of C$\bar{S}$H_2_ (d_hkl_ = 7.59, 4.28, 3.07, 2.87) were present in its diffractogram (Figure 1b), along with the reflections characteristic of anhydrite (C$\bar{S}$). The particle size distribution of the raw materials is presented in Figure 2.

**Figure 3:**
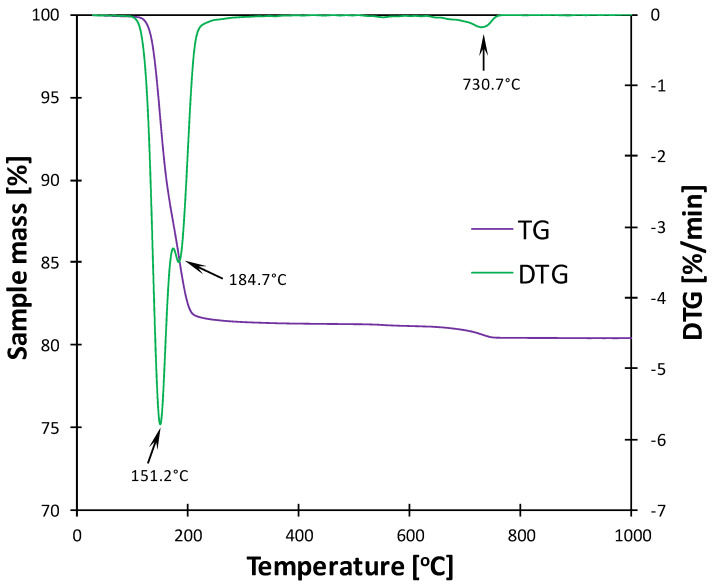
DTG/TG curves of gypsum used in the synthesis of calcium sulfoaluminate hydrates.

Additionally, thermal analysis was carried out to confirm the phase composition of gypsum (Figure 3). Two main endothermic effects were observed in the DTA/TG curves: the dehydration of C$\bar{S}$H_2_ and the formation of hemihydrate (C$\bar{S}$H_0.5_) at 151.2 °C, followed by the further loss of water and the transition of hemihydrate to anhydrite at 184.7 °C. Moreover, a minor effect attributed to the thermal decomposition of calcium carbonate was present at 730.7 °C; however, the associated mass loss (0.88 wt.%) was insignificant enough to declare the material appropriate for the synthesis of calcium sulfoaluminate hydrates. Stoichiometrically, gypsum contains 20.9 wt.% of water in its structure. Therefore, the obtained overall mass loss of 18.7 wt.% proves the high purity of CaSO_4_·2H_2_O used in the experiments and confirms the presence of a minor amount of anhydrite in the material.

The synthesis of calcium sulfoaluminate hydrates was carried out as follows. First, the C_3_A was ground to a specific surface area of 2800 cm^2^/g (acc. Blaine) in a planetary micro mill. Then, appropriate amounts of tricalcium aluminate and gypsum were mixed together and homogenized by sieving through a 125 µm mesh. Three different compositions of raw materials were prepared, characterized by the C_3_A:C$\bar{S}$ molar ratios of 1:1, 1:2 and 1:3. All of them were subsequently mixed with 250 mL of freshly distilled water (corresponding to a water/solid ratio of 50.0) and placed on magnetic stirrers inside a thermostatic water bath at a temperature of 23 °C. The samples were filtered through a Büchner funnel after 2 and 7 days of hydration. Afterwards, they were subjected to a two-step drying process. In the first step, whose goal was to remove the majority of free water, the samples were vacuum dried for 5 days at a pressure of 200 mbar using daily-replaced anhydrite-III as a desiccant. In the second step, the drying agent was changed to a supersaturated CaCl_2_ solution that provided a water vapor pressure of 200 mbar and a relative humidity of 35%. In these conditions, designed to remove the remaining free water while providing greater stability of the AFt and AFm phases (similar to the procedure described in [50]), the samples were dried to constant mass, i.e., to a daily mass change below 0.01%.

## 3. Results and Discussion

### 3.1. XRD Analysis

**Figure 4:**
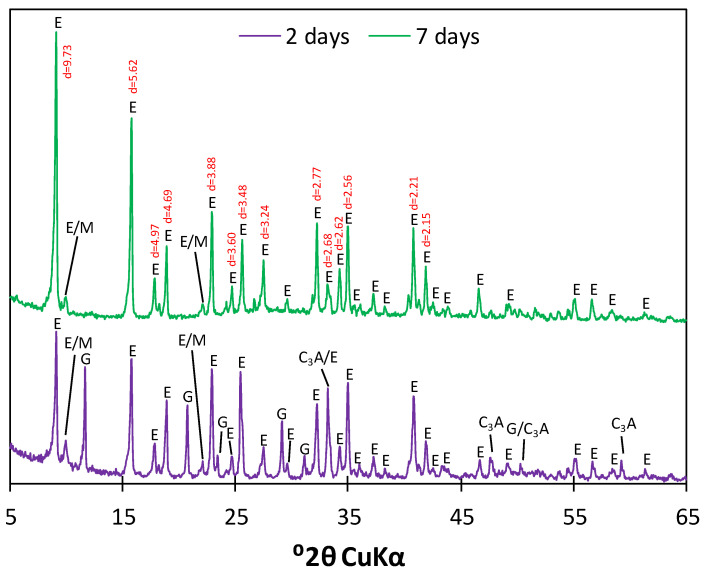
Diffractograms of the samples characterized by the C_3_A:C$\bar{S}$ molar ratio of 1:3 after 2 days (**purple**) and 7 days (**green**) of hydration; **E**—ettringite, **G**—gypsum, **M**—monosulfate.

After 2 and 7 days of hydration, samples characterized by the C_3_A:C$\bar{S}$ molar ratio of 1:3 (corresponding to ettringite) were subjected to the two-step drying process and analyzed by means of XRD to compare the changes in their phase composition related to the hydration progress. The obtained diffractograms are presented in Figure 4.

The XRD pattern of the sample hydrating for 2 days revealed the presence of numerous high-intensity reflections characteristic of unreacted gypsum (d_hkl_ = 7.59, 4.28, 3.07, 2.87) and tricalcium aluminate (d_hkl_ = 2.69, 2.21, 1.81). However, intensive peaks typical of ettringite were also present (d_hkl_ = 9.73, 5.62, 4.97, 4.69, 3.88, 3.60, 3.48, 3.24, 2.77, 2.69, 2.62, 2.56, 2.21, 2.15). In the diffractogram of the sample hydrating for 7 days, reflections characteristic of gypsum and tricalcium aluminate were not observed. Rietveld analysis estimated an ettringite content of 100% with a possible error of up to 2.9% (calculated as $\sqrt{n}$/n, where *n* is the number of measurement points) and goodness-of-fit (GOF) of 4.96. It has to be noted that a coincidence occurs between the main C_3_A peak and the ettringite peak at d_hkl_ = 2.69. Therefore, XRD analysis alone might be insufficient to determine whether the tricalcium aluminate present in the solution has reacted completely, especially considering that the detection limit for X-ray powder diffraction analysis can range from 0.2 to 5 wt.% [51]. Moreover, another coincidence occurs between monosulfate and ettringite peaks at d_hkl_ = 8.88 and 4.03, which can make the two phases difficult to separate if one of them is present only in trace amounts. Due to a higher hydration degree, 7-day samples were selected for further experiments.

**Figure 5:**
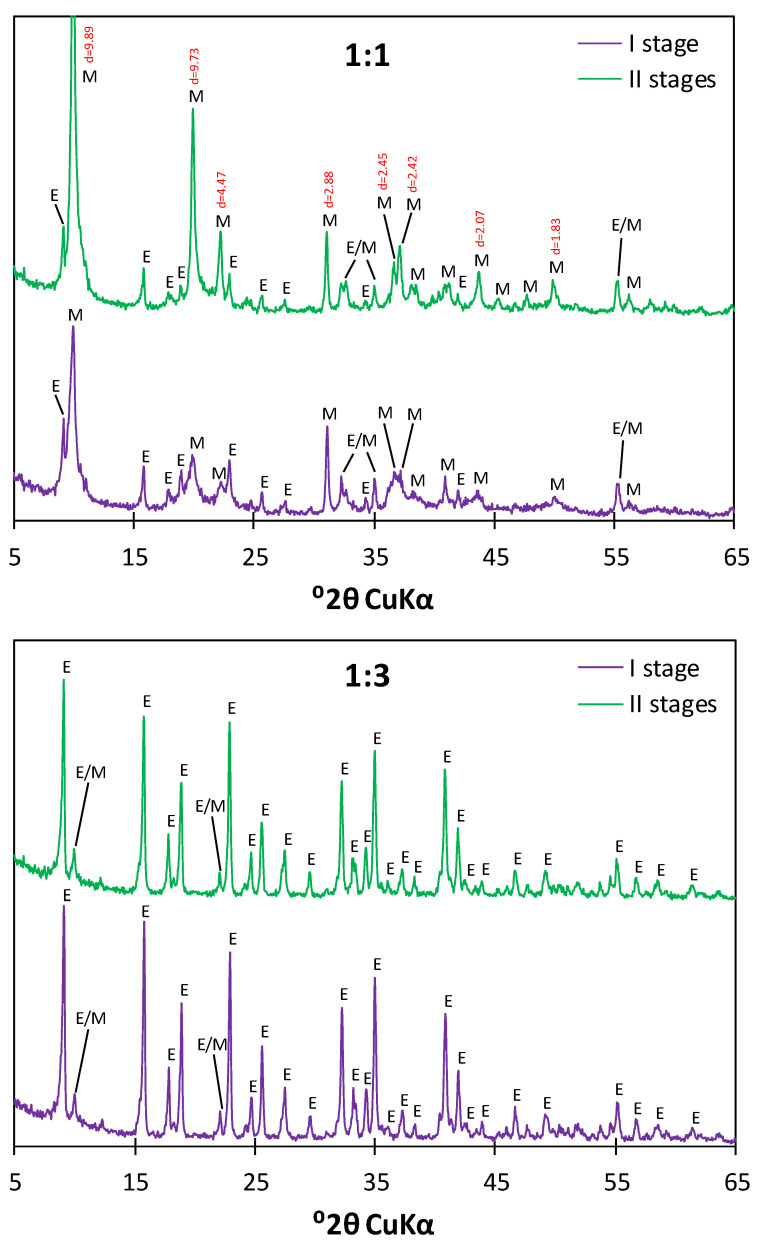
Diffractograms of the samples characterized by the C_3_A:C$\bar{S}$ molar ratios of 1:1 (**up**) and 1:3 (**down**) subjected to the one-step (**purple**) and two-step (**green**) drying processes; **E**—ettringite, **M**—monosulfate.

To determine the impact of the two-step drying method on the phase composition of hydrates, a comparison was made between the samples subjected only to the first step of drying (over anhydrite-III) and to both steps (over anhydrite-III and then supersaturated CaCl_2_). XRD analysis was carried out on samples characterized by the C_3_A:C$\bar{S}$ molar ratios of 1:1 and 1:3 (corresponding to the AFm and AFt phases, respectively). The obtained diffractograms are presented in Figure 5.

The obtained XRD patterns showed that, as expected, the main hydration products present in the samples characterized by the C_3_A:C$\bar{S}$ molar ratios of 1:1 and 1:3 were monosulfate and ettringite, respectively. In the case of the sample with the ratio of 1:1, the application of the two-step drying process resulted in a considerable increase in the intensity of reflections corresponding to the AFm phase (d_hkl_ = 9.89, 9.73, 4.47, 2.88, 2.45, 2.42, 2.07, 1.83). Such an outcome may be related to a decrease in the moisture of the sample and the transition of ettringite to monosulfate due to progress in hydration, but also to the increased stability of monosulfate resulting from a higher water vapor pressure over the CaCl_2_ solution. In the case of the samples characterized by the ratio of 1:3, no significant difference was found between the patterns of the specimens subjected to one-step and two-step drying processes. The additional drying step had no visible impact on the intensity of the peaks corresponding to ettringite. Therefore, the two-step drying technique, providing better stability of the obtained phases, was selected for further experiments focused on the characterization of the calcium sulfoaluminate hydrates.

**Figure 6:**
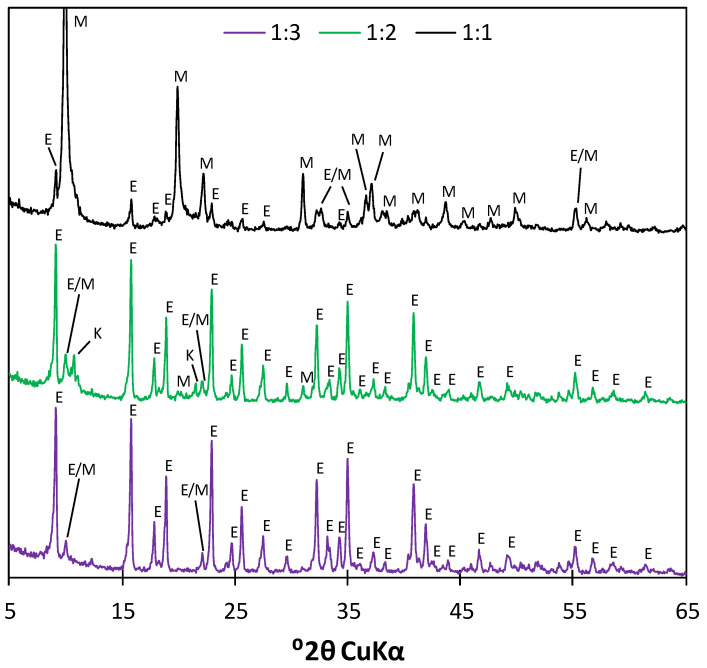
Diffractograms of the samples characterized by the C_3_A:C$\bar{S}$ molar ratios of 1:1 (**black**), 1:2 (**green**) and 1:3 (**purple**) subjected to the two-step drying process after 7 days of hydration; **E**—ettringite, **M**—monosulfate, **K**—carboaluminate.

The diffractograms of the samples characterized by all three C_3_A:C$\bar{S}$ molar ratios, subjected to the two-step drying process after 7 days of hydration, are presented in Figure 6.

According to the obtained XRD patterns, the sample characterized by the C_3_A:C$\bar{S}$ molar ratio of 1:1 contained mostly monosulfate (72.1%, acc. Rietveld analysis; GOF of 6.08), as proved by the presence of reflections corresponding to the d-spacing of d_hkl_ = 8.93, 4.47, 4.00, 2.88, 2.45, 2.42, 2.07 and 1.83. Ettringite was present in a lower amount (27.9%). Meanwhile, as mentioned earlier, for the C_3_A:C$\bar{S}$ ratio of 1:3, ettringite was the only phase detected during the Rietveld analysis (d_hkl_ = 9.73, 5.62, 4.97, 4.69, 3.88, 3.60, 3.48, 3.24, 2.77, 2.69, 2.62, 2.56, 2.21 and 2.15, GOF of 4.96). However, due to the coincidence of AFm and AFt reflections at d_hkl_ = 8.88 and 4.03, it is possible that a minor amount of monosulfate was present in the sample. In the case of the sample with the 1:2 molar ratio, peaks typical of both aforementioned phases were observed, with a higher intensity of those corresponding to ettringite. The approximate ettringite and monosulfate contents (acc. Rietveld analysis) were 59.6% and 40.4%, respectively, with a GOF of 6.67. Additionally, traces of hemicarboaluminate hydrate (main peak at 10.8 °2θ, d_hkl_ = 8.20) were found in the pattern (ref. code 041-0221). The phase was most likely formed as a result of sample carbonation that occurred during its preparation.

Based on the obtained XRD patterns, crystallographic characteristics of the synthesized ettringite (ref. code 98-002-7039) and monosulfate (ref. code 98-010-0138) were derived from the ICSD database cards and are presented in Table 2 and Table 3.

### 3.2. Thermal Analysis

**Figure 7:**
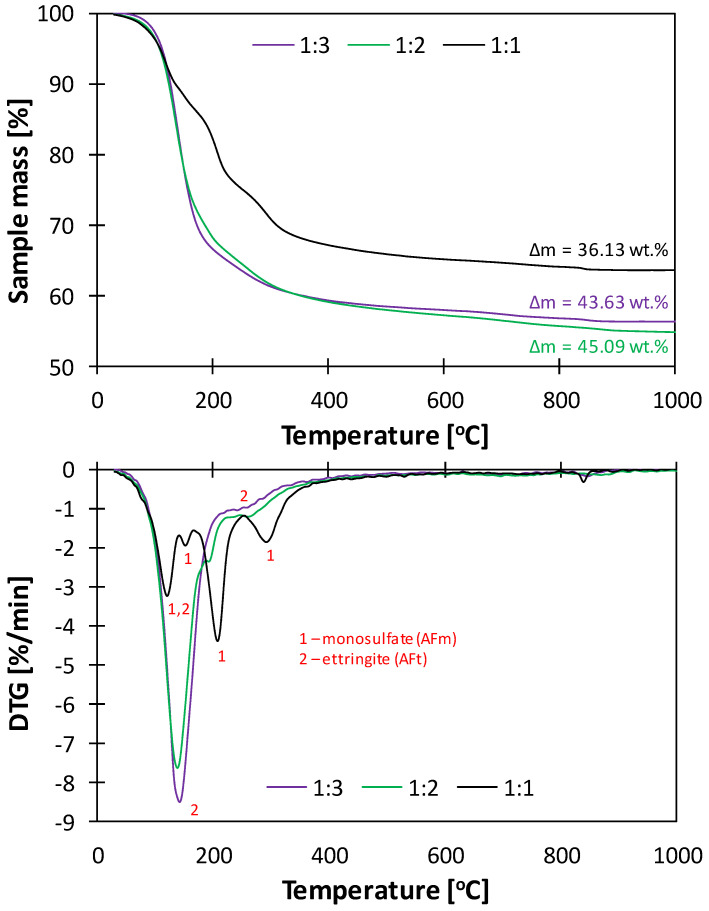
TG/DTG curves of the samples characterized by the C_3_A:C$\bar{S}$ molar ratios of 1:1 (**black**), 1:2 (**green**) and 1:3 (**purple**) subjected to the two-step drying process after 7 days of hydration.

The DTA/TG curves of the samples characterized by all three C_3_A:C$\bar{S}$ molar ratios, subjected to the two-step drying process after 7 days of hydration are presented in Figure 7.

According to the literature, the thermal decomposition of monosulfate is a four-step process [52]. Consequently, four endothermic effects were found at approx. 130, 153, 209 and 293 °C in the DTG curve of the sample characterized by the C_3_A:C$\bar{S}$ molar ratio of 1:1. However, the first effect occurs in the same temperature range for both ettringite and monosulfate (130–150 °C); therefore, the observed peak was coincidental for the two phases. Moreover, the peak at 293 °C can also be assigned to the dehydroxylation of amorphous AH_3_ (colloidal aluminum hydroxide), which can be formed as one of the C_3_A hydration products. Additionally, a minor endothermic effect was present at approx. 850 °C, most likely related to the decomposition of the carboaluminate phase formed due to sample carbonation. The DTG curve of the specimen characterized by the ratio of 1:3 showed only one pronounced effect with a maximum at 140 °C, consistent with the presence of ettringite. It is, however, possible that trace amounts of monosulfate were hidden in the TG/DTG curves due to the aforementioned peak coincidence. In the DTG curve of the sample with the C_3_A:C$\bar{S}$ ratio of 1:2, a pronounced effect corresponding to ettringite with a maximum at approx. 140 °C was observed, though the associated mass loss was slightly lower than in the case of the specimen with the ratio of 1:3. In addition, a minor peak related to the thermal decomposition of monosulfate was present at approx. 200 °C.

Due to the observed coincidences in the DTG/TG curves, it is impossible to clearly separate the endothermic effects originating from monosulfate, ettringite and AH_3_, which poses a limitation of the method. Therefore, in order to better evaluate the purity of the obtained AFm and AFt phases, experimentally obtained mass loss related to the dehydration of the samples was compared with the stoichiometric amounts of water present in the structures of ettringite and monosulfate (Table 4).

The experimentally determined chemically bound water contents are close to the stoichiometric values calculated for ettringite and monosulfate. In the case of the sample characterized by the C_3_A:C$\bar{S}$ molar ratio of 1:1, the obtained mass loss was 1.42% higher than the stoichiometric water content in monosulfate. Such a result correlates well with the outcome of the XRD analysis, which indicated the presence of minor amounts of ettringite in the material. Analogously, the sample with the C_3_A:C$\bar{S}$ ratio of 1:3 exhibited a 2.25% lower mass loss compared to the amount of water present in the structure of ettringite, most likely due to the presence of monosulfate traces or partial ettringite dehydration prior to the analysis.

Based on the obtained results, it can be concluded that the selected synthesis method yielded mostly monosulfate at the C_3_A:C$\bar{S}$ molar ratio of 1:1 and ettringite at the ratio of 1:3. However, if chemically pure monosulfate needs to be obtained, a different synthesis technique has to be found.

### 3.3. FTIR Spectroscopy

**Figure 8:**
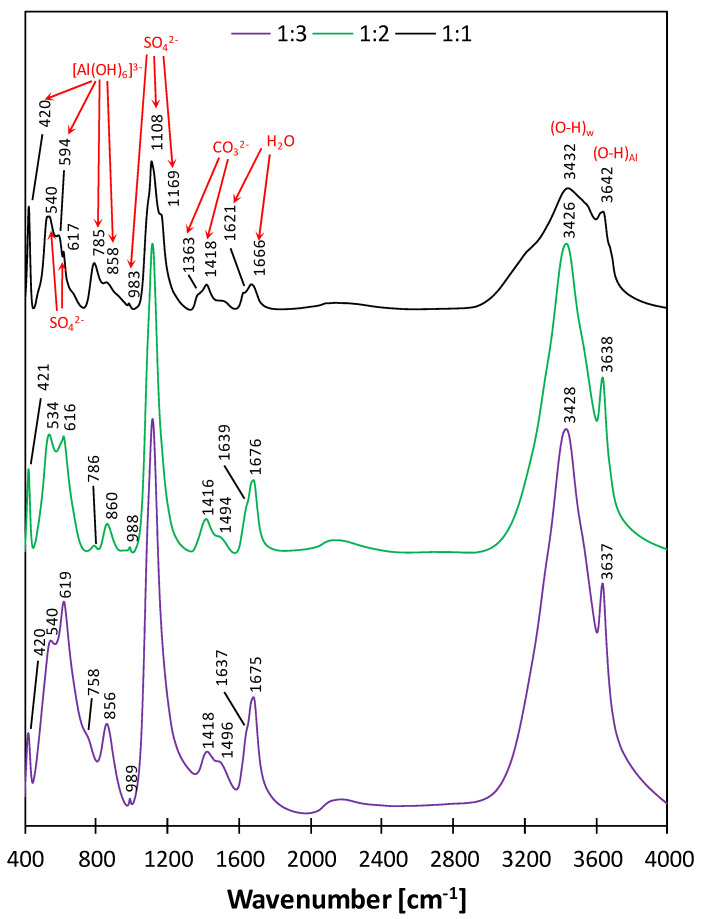
FTIR spectra of the samples characterized by the C_3_A:C$\bar{S}$ molar ratios of 1:1 (**black**), 1:2 (**green**) and 1:3 (**purple**) subjected to the two-step drying process after 7 days of hydration.

The FTIR spectra of the samples with various C_3_A:C$\bar{S}$ molar ratios are presented in Figure 8. Additionally, the positions of particular absorption bands, along with their possible interpretations, are given in Table 5.

In the structure of ettringite, O-H bonds are present in [Al(OH)_6_]^3−^ octahedrons and in water molecules. In the wavenumber range of 3650–3400 cm^−1^, attributed to the highest energy bonds, the presence of two bands was observed in the spectra of all three samples. The narrow band at approx. 3640 cm^−1^ can be assigned to the O-H stretching vibrations (both symmetrical and asymmetrical) in [Al(OH)_6_]^3−^ [53]. The wider band at approx. 3430 cm^−1^ was assigned to asymmetrical O-H stretching vibrations in H_2_O molecules [52]. In the case of the samples with the C_3_A:C$\bar{S}$ ratios of 1:2 and 1:3, both bands were characterized by a smaller full width at half-maximum and higher intensities compared to the ratio of 1:1. The effect can be attributed to a higher ordering degree of the water molecules present in the ettringite structure compared to monosulfate. In the 1:1 sample, the ordering degree was lower, which means that the wider band originated from the H_2_O molecules present within the AFm crystal structure. The AFm hydrates, which belong to the LDH family (layered double hydroxides, structurally related to portlandite–Ca(OH)_2_), consist of cationic layers of Ca_2_Al(OH)_6_^+^, separated by interlayers containing anions surrounded by water molecules [50].

In all three spectra, the next two absorption bands were observed in the range of 1670–1620 cm^−1^. Based on [53,54], they were assigned to the bending vibrations of H-O-H bonds in H_2_O molecules. Additionally, bands related to the stretching vibrations of C-O bonds present in carbonate ions (CO_3_^2−^) were revealed in the range of 1300–1500 cm^−1^ [55]. During the synthesis of the samples, various precautions were taken to protect them from carbonation. Nevertheless, in the case of two specimens, the XRD and TG/DTG analyses proved the formation of carboaluminate phases. In AFm compounds, SO_4_^2−^ anions can be substituted by CO_3_^2−^ due to their specific LDH structure. Carboaluminate hydrates are more stable than monosulfoaluminate hydrates and therefore, when in-air CO_2_ is available, they create solid solutions [18]. However, the FTIR spectra revealed only low-intensity bands assigned to the C-O bonds, which shows that the carboaluminate phase was formed only in minor amounts.

In the wavenumber range of 1200–980 cm^−1^, bands characteristic of SO_4_^2−^ ions were observed. The main band, at approx.. 1110 cm^−1^, was assigned to the asymmetrical stretching vibrations of S-O bonds in ettringite [53,56]. In the case of the sample with the C_3_A:C$\bar{S}$ ratio of 1:1, the position of the band had switched to 1169 cm^−1^, which corresponds to the differences in the structures of ettringite and monosulfate. The bands at about 990 cm^−1^ are related to the symmetrical stretching vibrations of S-O bonds [53]. Furthermore, in all three spectra, two bands appeared in the range of 860–780 cm^−1^ that were attributed to the bending vibrations of Al-O-H bonds present in the [Al(OH)_6_]^3−^ octahedrons found in ettringite and monosulfate [53]. Moreover, for all samples, the absorption bands at about 618 cm^−1^ and 540 cm^−1^ were assigned to the stretching vibrations of S-O bonds, with the first one being the most intensive in the case of the sample with the C_3_A:C$\bar{S}$ ratio of 1:3 due to its higher ettringite content. The presence of Al in octahedral coordination was confirmed by the band at approx. 594 cm^−1^ [57]. The band at about 420 cm^−1^ can be attributed to the bending vibrations of the Al-O-H and O-Al-O bonds [53]. The Ca-O bonds that are present in the structure of both ettringite and monosulfate were not observed in the spectra, as they are characterized by wavenumbers that are below 400 cm^−1^.

## 4. Conclusions

The presented study was focused on the synthesis and characterization of hydrated calcium sulfoaluminates—monosulfate and ettringite. Developing a method for the synthesis of pure AFm and AFt phases—the main hydration products in CSA and BYF cement systems—is crucial for further research on sustainable cementitious composites that contain these binders, especially in the case of studies focused on the durability of hydrates in different corrosive environments. Based on the obtained results, the following conclusions were drawn:

- The synthesis of calcium sulfoaluminate hydrates, carried out from tricalcium aluminate and gypsum in an excess amount of water (w/s ratio of 50.0) over a 7-day period, yields ettringite and monosulfate as the main products—no traces of the starting materials (C_3_A and gypsum) were found during the analyses.
- Thermal analysis, X-ray diffractometry and FTIR spectroscopy can be used to distinguish the characteristic features of synthesized ettringite and monosulfate.
- According to Rietveld analysis, the synthesis carried out at a C_3_A/C$\bar{S}$ molar ratio of 1:3 yields pure ettringite. In the case of the sample characterized by the ratio of 1:1 (typical of monosulfate), a considerable portion of ettringite (27.9%) was present in the final products along the AFm phase. Therefore, a different synthesis method has to be selected in order to obtain pure monosulfate, such as precipitation from a solution containing Al_2_(SO_4_)_3_·18H_2_O and Ca(OH)_2_ mixed at an appropriate molar ratio. Moreover, suitable conditions (i.e., the selection of a drying agent that offers sufficient water vapor pressure and prevention from contact with in-air CO_2_ both during and after the synthesis) have to be maintained in order to ensure the stability of the monosulfate phase.
- The dehydration of monosulfate occurs in four steps, with the endothermic effects observed at approx. 130, 153, 209 and 293 °C. Such multistage thermal decomposition can also be related to the presence of trace amounts of ettringite, hemicarboaluminate or amorphous aluminum hydroxide in the sample. Dehydration of ettringite is a single-step process, with the endothermic effect observed in the temperature range of 130–150 °C.
- The obtained FTIR spectra of monosulfate and ettringite show distinctive features related to the differences in their crystal structures that allow distinction between the AFm and AFt phases with the use of spectroscopic methods. Both phases consist of [Al(OH)_6_]^3−^ octahedrons, SO_4_^2−^ anions and H_2_O molecules. The variations in their structure are evidenced by the changes in the mutual intensity ratios of the bands observed in the range of 860–780 cm^−1^, assigned to the bending vibrations of Al-O-H bonds. Additionally, a significant difference is observed in the intensity of the absorption bands at approx. 1169, 1108, 619 cm^−1^ and 540 cm^−1^, assigned to the vibrations of S-O bonds. In the spectrum of the sample containing mainly monosulfate, an additional band appeared at approx. 1169 cm^−1^, while in the case of the sample consisting of ettringite, the band at about 619 cm^−1^, related to the stretching vibrations of S-O bonds, was considerably more intensive compared to the other two samples. The wide band at approx. 3430 cm^−1^, assigned to asymmetrical stretching vibrations of O-H in water molecules, was also more intensive in the case of ettringite-containing samples, which proved a higher content of water bound in the structure of the phase compared to monosulfate.

## Figures and Tables

**Table 1:** Phase composition of the reaction products of C_3_A and C$\bar{S}$H_2_ mixed at a molar ratio of 1:1 in an excess amount of water [32].

Hydration Time	Phase Composition of the Products at 30 °C	Phase Composition of the Products at 50 °C
30 min	Gypsum + C_3_A + small amounts of ettringite	Gypsum + C_3_A + small amounts of ettringite
3 days	Ettringite + C_3_A + traces of gypsum	Ettringite + C_3_A + traces of gypsum
14 days	Ettringite + traces of C_3_A	Monosulfate + ettringite
28 days	Ettringite + monosulfate	Monosulfate
60 days	Monosulfate	Monosulfate + traces of ettringite
90 days	Monosulfate + traces of ettringite	Monosulfate + ettringite

**Table 2:** Crystallographic parameters of the obtained synthetic ettringite.

**Crystal System**	**Hexagonal**
**Space Group**	**P63/mcm**
**Cell Parameters**	
**a**	11.230
**b**	11.230
**c**	10.720
**Density [g/cm^3^]**	1.78
**Unit cell volume [10^6^ pm^3^]**	1170.81

**Table 3:** Crystallographic parameters of the obtained synthetic monosulfate.

**Crystal System**	**Hexagonal**
**Space Group**	**R-3**
**Cell Parameters**	
**a**	5.759
**b**	5.759
**c**	26.795
**Unit cell volume [10^6^ pm^3^]**	769.62

**Table 4:** Comparison of the thermal analysis results with the stoichiometric water content in ettringite and monosulfate.

	**Stoichiometric Water Content [%]**	
	**Monosulfate**	**Ettringite**
	34.71	45.91
$C_{3}A:C\bar{S}$ **molar ratio**	**1:1**	**1:3**
**TG mass loss [%]**	36.13	43.63

**Table 5:** Interpretation of the absorption bands present in the FTIR spectra.

Absorption Band [cm^−1^]	Possible Assignment	Interpretation
3642–3637	ν_s_ (OH)_Al_	[Al(OH)_6_]^3−^
3432–3428	ν_as_ (OH)_w_	H_2_O
1676–1621	δ (H-O-H)	H_2_O
1496–1363	ν C-O	CO_3_^2−^
1169	ν_as_ S-O	SO_4_^2−^ (monosulfate)
1115, 1113, 1108	ν_as_ S-O	SO_4_^2−^ (ettringite)
989, 988, 983	ν_s_ S-O	SO_4_^2−^
860–616	δ Al-O-H	[Al(OH)_6_]^3−^
594	Octahedral Al	[AlO_6_] in AFm
540–534	ν S-O	SO_4_^2−^ (ettringite)
421–420	δ Al-O-H δ O-Al-O	[Al(OH)_6_]^3−^

## Data Availability

The raw data supporting the conclusions of this article will be made available by the authors on request.

## Abbreviations

Cement chemistry notation is used throughout the manuscript to describe the mutual molar ratios of oxides:

C	CaO
S	SiO_2_
A	Al_2_O_3_
F	Fe_2_O_3_
$\bar{S}$	SO_3_
$\bar{C}$	CO_2_
H	H_2_O
